# SCMR President's Page

**DOI:** 10.1186/1532-429X-13-1

**Published:** 2011-01-05

**Authors:** Eike Nagel

**Affiliations:** 1 King's College London, Division of Imaging Sciences, The Rayne Institute | 4th Floor Lambeth Wing, St. Thomas' Hospital | London SE1 7EH

## 

Many of you will now slowly start thinking about 2011. The effort of putting abstracts together is finished (with a record number of abstracts submitted) and we are in the final stages of planning the 2011 SCMR/Euro CMR Joint Scientific Sessions to be held in Nice February 4-6, 2011.

I am happy to report on the latest activities from the SCMR Board of Trustees. We held our mid-year meeting in Philadelphia at the Airport Hotel. One of the key tasks of this Board meeting is to examine the 2011 scientific program to find and eliminate any weaknesses or risks. In addition, the work of the organization's committees is reported and reviewed, the platinum supporters and the Board have an open discussion, and the members of the Board review the management of the Society.

2011 Program Chair Sven Plein MD and Abstract Chair Raymond Kwong MD have put a fantastic program together. There are a couple of new features this year. For PhDs there is a basic science preconference on pulse sequence design, a translational abstract track, extended poster viewing times and early morning sessions on basic cardiology. For Physicians there is a full parallel track with cases and how-to sessions as well as early morning sessions on basic physics. For congenital heart disease specialists there is a one-day preconference, and more cases and how-to sessions. Technologists will enjoy full access to the meeting and can join the cases and how-to sessions. All this, on top of the outstanding features of the previous conferences.

We anticipate even more participants than the record meeting last year and believe that we have listened to and integrated the wishes of our membership and attendees. We are looking forward to a meeting in Europe with more European participants, speakers, and influence then ever. We also hope to strengthen our ties with France and welcome many French attendees. To improve the access to knowledge on CMR in France, a Wikipedia webpage has been developed in French (led by Mark Westwood MD and his French collaborators).

The "Young Investigator Award" has been renamed to "Early Career Award" to clearly state that investigators who had a late start in CMR can also achieve this award.

SCMR will again offer Regional Scholarships for the 2011 meeting. This year for the first time, a Technologist Scholarship will be awarded.

The SCMR 2012 meeting will take place at the Marriott World Center in Orlando (2/2 - 2/5/2012), SCMR 2013 will be held at the San Francisco Hilton, and we will start the discussion on the location of the 2014 scientific sessions immediately after the Nice meeting.

In addition to the joint meeting, other collaborations with the Euro CMR Working Group include efforts to harmonize the training guidelines (an update has been submitted by Sven Plein MD). SCMR is working with the European Working Group to re-write the indications paper, and the Euro CMR registry (Oliver Bruder MD and Heiko Mahrholdt MD) now includes patients throughout Europe.

The SCMR Board has approved the formation of a Middle East Study Group, open to all countries in the Middle East.

Our Journal has further improved its impact factor from 2.15 to 2.28. This reflects both the work of the scientists submitting their excellent work to *the Journal of Cardiovascular Magnetic Resonance *and the efforts of our editor Dr. Dudley Pennell MD and his team.

The Website has changed considerably over the summer. We have now implemented the "single sign-on" which allows all members to log-in once and then access all members-only content and the secure registration/membership pages without repetitive sign-in. The Case of the Week section is continuously expanding and will soon be complemented with a forum for feedback. The user interface will be redesigned to improve functionality and speed for finding relevant information, and an online environment for training in CMR will be available in the near future. This work is continuously pushed forward and organized by our Web Editor James Moon MD.

We are planning a two-day training course in CMR to be held at the NIH in spring 2011. Details will be posted on the SCMR website as they become available.

In addition to the standardized data acquisition (citation) and reporting guidelines (citation) we are currently working on standardized post-processing for further harmonization and improved reproducibility between sites. Jeanette Schulz-Menger MD leads this effort.

SCMR's membership has increased 10% since last year and its finances are stable. We have implemented a new category of membership (assisted membership) available to applicants from developing countries. The details of this membership will be available on the website soon.

It has been a good year for SCMR with many positive developments. This is due to our members, your Board of Trustees, the SCMR committees, and our Management team led by Deborah Berkowitz.

